# A Highly Efficient Recombinant Laccase from the Yeast *Yarrowia lipolytica* and Its Application in the Hydrolysis of Biomass

**DOI:** 10.1371/journal.pone.0120156

**Published:** 2015-03-17

**Authors:** Dayanand Kalyani, Manish Kumar Tiwari, Jinglin Li, Sun Chang Kim, Vipin C. Kalia, Yun Chan Kang, Jung-Kul Lee

**Affiliations:** 1 Department of Chemical Engineering, Konkuk University, Seoul, Korea; 2 Department of Biological Sciences, Korea Advanced Institute of Science and Technology, Yuseong-gu, Daejeon, Korea; 3 Microbial Biotechnology and Genomics, CSIR-Institute of Genomics and Integrative Biology, Delhi University Campus, Delhi, India; 4 Department of Materials Science and Engineering, Korea University, Anam-Dong, Seongbuk-Gu, Seoul, Korea; Queen's University Belfast, UNITED KINGDOM

## Abstract

A modified thermal asymmetric interlaced polymerase chain reaction was performed to obtain the first yeast laccase gene (YlLac) from the isolated yeast *Yarrowia lipolytica*. The 1557-bp full-length cDNA of YlLac encoded a mature laccase protein containing 519 amino acids preceded by a signal peptide of 19 amino acids, and the YlLac gene was expressed in the yeast *Pichia pastoris*. YlLac is a monomeric glycoprotein with a molecular mass of ~55 kDa as determined by polyacrylamide-gel electrophoresis. It showed a higher catalytic efficiency towards 2,2-azino-bis(3-ethylbenzothiazoline-6-sulfonate) (*k_cat_/K_m_* = 17.5 s^-1^ μM^-1^) and 2,6-dimethoxyphenol (*k_cat_/K_m_* = 16.1 s^-1^ μM^-1^) than other reported laccases. The standard redox potential of the T1 site of the enzyme was found to be 772 mV. The highest catalytic efficiency of the yeast recombinant laccase, YlLac, makes it a good candidate for industrial applications: it removes phenolic compounds in acid-pretreated woody biomass (*Populus balsamifera*) and enhanced saccharification.

## Introduction

Laccase (benzenediol: oxygen oxidoreductase, EC 1.10.3.2) is the most abundant member of the multicopper protein family that catalyses the oxidation of substituted phenols using molecular oxygen as a final electron acceptor [1]. Laccase is one of the oldest enzymes reported and is currently eliciting great interest in the scientific community because of its industrial importance and huge catalytic capabilities, making it one of the ‘‘greenest” enzymes of the 21^st^ century [2]. Laccases are widely distributed in nature, occurring in fungi as well as in plants, insects, bacteria and archaea [1,3]. In fungi, laccases are mainly produced by the deuteromycetes, ascomycetes, and basidiomycetes and are involved in plant pathogenesis, pigmentation, detoxification, and lignin degradation [4]. Because of their surprisingly wide variety of substrates, laccases are useful biocatalysts for a wide range of biotechnological application such as decolorization of industrial dyes and textile dye effluents, delignification of lignocellulosic biomass, detoxify pulp bleaching, determination of bilirubin levels in serum, juice and wine clarification, transformation of antibiotics and steroids [5,6]. In addition, laccases have demonstrated potential for use in biosensors, bioreactors, and biofuel cell [7].

In an attempt to obtain significant amounts of enzyme for biotechnological applications and to use laccase more efficiently in biotechnology, several laccase genes have been cloned from different microorganisms and heterologously expressed in yeasts (*Saccharomyces cerevisiae*, *Pichia pastoris*, *Pichia methalonica*, *Yarrowia lipolytica* and *Kluyveromyces lactis*), filamentous fungi (*Aspergillus niger*, *Aspergillus oryzae* and *Trichoderma reesei*) and bacteria such as *Escherichia coli* and *Streptomyces lividans* [8,9]. The promising and valuable applications of laccase in biotechnology and industry have resulted in increased interest and a need for the isolation of new laccase genes from different sources [10]. In addition, the isolation of new laccase genes from novel microbes will greatly promote the precise elucidation of the biological function of laccase. To our knowledge, the isolation and characterization of laccase gene from yeast species has never been reported.

Conversion of abundant lignocellulosic biomass to biofuels as transportation fuels presents a viable option for improving energy security and reducing greenhouse emissions. The digestibility of cellulose present in lignocellulosic biomass is hindered by many physicochemical, structural, and compositional factors. Pretreatment of biomass plays a critical role in producing materials with acceptable enzymatic digestibility and subsequent fermentability for the production of cellulosic ethanol or other advanced biofuels. Steam explosion, a process that combines high pressures and temperatures, is one of the most commonly used pretreatment methods, which is especially effective for hardwoods and agriculture crops [11]. During the pretreatment of lignocellulosic feedstock to produce fermentable sugars, causes various structural alterations inside the lignocellulosic material. Lignin is redistributed and hemicellulose is partially hydrolyzed and solubilized, making cellulose more accessible to enzymes [12]. In contrast, this pretreatment generates some soluble inhibitory components, which can affect enzymatic hydrolysis as well as fermentation steps [12,13,14]. Therefore, the facile removal of these inhibitory compounds would aid subsequent enzymatic hydrolysis and improve the overall sugar and fermentation process.

Here, we describe for the first time the functional expression of a highly efficient yeast laccase from the previously isolated yeast *Yarrowia lipolytica* SKU507 [15]. The yeast laccase gene YlLac and its corresponding full-length cDNA were cloned and characterized. The YlLac gene was successfully expressed in the yeast *P*. *pastoris*, which confirmed the correct function of the laccase encoded by the YlLac gene. As a demonstration of its potential industrial application, the YlLac enzyme was used to detoxify biomass (*Populus balsamifera*) to enhance its saccharification by cellulase.

## Materials and Methods

### Microbial strains and media

The strains, primers, and plasmids used are summarized in Table 1. *Y*. *lipolytica* SKU507 was isolated from soil samples collected from Sorak Mountain, Republic of Korea [15], and was deposited at the Korean Culture Center of Microorganisms (KCCM 11502P). The strain was sub-cultured every 3 weeks and stored at 4°C on potato dextrose agar (PDA) plates. *Escherichia coli* DH5α competent cells were used for subcloning procedures and were grown in Low Salt LB medium. *Pichia pastoris* KM71H was products of Invitrogen (Carlsbad, CA, USA). Yeast extract-peptone-dextrose (YPD), buffered glycerol-complex (BMGY) and buffered minimal methanol (BMM) media were prepared according to the manual of the EasySelect Pichia Expression Kit (Invitrogen).

**Table 1:** Strains, plasmids, and oligonucleotide primers used in this study.

Strains, plasmid or primer	Description	Source or reference
Strains		
*E*. *coli* DH5α	F^−^ *ɸ80dlacZΔM15 Δ(lacZYA-argF)*U169 *recA1 endA1 hsdR17* (rk-, mk+) *supE44 thi-1 gyrA relA1*	Life Technologies
*Y*. *lipolytica*	Strain SKU507; isolated from soil sample	[15]
*P*. *pastoris* KM71H	Expression host. *aox1*:*ARG4*,*arg4*	Life Technologies
Plasmids		
pGEM-T-Easy	*ori* pMB1, Amp^r^	Promega
pPICZαA	P. pastoris 3.6-kb protein expression and secretion vector carrying a methanol-inducible promoter (PAOX1), 5′ AOX1 region, MFα1_s_; Zeo^r^	Invitrogen
pPICZαA-Sp-YlLac	5′AOX1 region; Zeo^r^; with YlLac for expression in P. pastoris	This study
pPICZαA-Wsp-YlLac	5′AOX1 region, MFα1_s_; Zeo^r^; with YlLac for expression in P. pastoris	This study
Primers (5′-3′)		
CuI	CAYTGGCAYGGNTTYTTYCA	[16]
CuIV	TGRAARTCDATRTGRCARTG	
SPR1	AGGTGGCTATGG TACCAGAACGTTCCAGC	This study
SPR2	GCAGCAGTGAAGTCGTATAGGAACGAGTTG	This study
SPR3	GGACACTGGTTGACGAAAGCAGGTCCGTCC	This study
SPF1	CGTCCTTGGCTGCAGACGGACAATCCGG	This study
SPF2	TGTTCACACACATATCTATCTCCCGGCCGC	This study
SPF3	CGGCACGGCTGGCGACAATGTCACCATCCG	This study
YlLac-f1	ATGAACTTTGTGACCGCACTCCCACTG	This study
YlLac-r1	TTGCAGATCCGGGCCTAAACGTCCACG	This study
Sp-YlLac-f1	*ctc*gagATGAACTTTGTGACCGCACTCCCAC^*a*^	This study
Sp-YlLac-r1	*tct*agaTTGCAGATCCGGGCCTAAACGTCCAC^*b*^	This study
Wsp-YlLac-f1	*ggt*accGCCATTGGCCCGGTGACC^*c*^	This study
Wsp-YlLac-r1	*tct*agaTTGCAGATCCGGGCCTAAACGTCCAC^*b*^	This study
AD1	NTGCANTNTGCNGTT	[17]
AD2	NGTCAGNNNGANANGAA	
AD3	NGTGNGANANCANCANAG	
AD4	TGNGNGANANCANAG	
AD5	AGNGNAGNANCANAGC	

^*a*^The XhoI site is in small letters.

^*b*^The XbaI site is in small letters.

^*c*^The KpnI site is in small letters.

W = A / T, S = G / C, N = A/T / G / C

### Chemicals

2, 2′-Azino-bis (3-ethylbenzthiazoline-6-sulfonic acid) (ABTS), 2,6-dimethoxyphenol (DMP), guaiacol, toluidine, 3-aminobenzoic acid and phenyldiamine were purchased from Sigma-Aldrich (St. Louis, MO), respectively. All other chemicals and reagents were of analytical grade and were purchased from commercial sources, unless otherwise stated. Reagents for polymerase chain reaction (PCR), Ex-Taq DNA polymerase, the genomic DNA extraction kit, and the pGEM-T easy vector were purchased from Promega (Madison, WI). T4 DNA ligase and restriction enzymes were obtained from New England Biolabs (Beverly, MA). Plasmid pPICZαA and Zeocin were purchased from Invitrogen (Carlsbad, CA).

### Cloning of the complete structural gene of YlLac

The degenerate primers CuI and CuIV (Table 1) were designed based on the conserved amino acid sequences of copper-binding regions I (HWHGFFQ) and IV (HCHIDFH) and partial peptide sequences obtained from nano-LC-MS/MS sequencing [14]. Degenerate PCR was performed using the genomic DNA of *Y*. *lipolytica* SKU507 as the template. A 1523-bp PCR fragment was obtained and cloned into the pGEM-T Easy Vector for sequencing. DNA sequencing confirmed that this 1523-bp PCR fragment contained a sequence for a laccase gene. In order to obtain the complete structural gene encoding laccase, thermal asymmetric interlaced PCR (TAIL-PCR) [18] was performed to amplify the 5′ and 3′ sequences flanking the known partial sequence of the 1523-bp using the genomic DNA of *Y*. *lipolytica* SKU507 as the template. Three interlaced specific primers, namely SPR1, SPR2, and SPR3, and SPF1, SPF2 and SPF3, were used for flanking the 5′ and 3′ regions, respectively (Table 1). Five random degenerate primers (RP), AD1, AD2, AD3, AD4, and AD5 (Table 1), in which N represents A/G/C/T, were used for both the flanking 5′ and 3′ regions. The TAIL-PCR product was cloned into the pGEM-T Easy Vector for DNA sequencing. After obtaining the 5′ and 3′ flanking sequences of the known 1523-bpsequence, high-fidelity PCR was performed to amplify the complete structural gene using the genomic DNA of *Y*. *lipolytica* SKU507 as the template and YlLac-f1 and YlLac-r1 as the specific primers (sequences shown in Table 1). The PCR product, the 1859-bp complete structural gene encoding laccase, was cloned into the pGEM-T Easy Vector (Promega) for DNA sequencing and designated as YlLac.

### Isolation and sequencing of cDNAs

Total RNAs were extracted using QIAGEN RNeasy Plant kit (QIAGEN, Italy) following the manufacturer’s instructions. Based on the known 5′- and 3′-end sequences of the laccase structural gene, the primer Yllac-f1 was designed to complement the start codon (ATG) region and the primer Yllac-r1 was designed to complement the sequence immediately downstream of the stop codon Table 1). Using Yllac-f1 and Yllac-r1 as the specific primers, RT-PCR was then performed to amplify the full-length cDNA using PrimeSTAR HS DNA Polymerase (Takara Bio, Shiga, Japan). The amplified cDNA fragments were then eluted and cloned, and their identities were confirmed by sequencing.

### DNA manipulations and gene sequence analysis

Analysis of the homology between the protein encoded by YlLac and other known laccase proteins was performed using the BLAST program (http://blast.ncbi.nlm.nih.gov/Blast.cgi). The molecular weight and isoelectric point of protein were predicted using the Compute pI/Mw tool (http://www.expasy.org/tools/pi_tool.html). N-glycosylation sites (Asn-X-Ser/Thr) were identified using the ScanProsite program (http://www.expasy.ch/tools/scanprosite/). Signal peptides were predicted using SignalP 3.0 (http://www.cbs.dtu.dk/services/SignalP/). The conserved domains of the protein were predicted, and they were analyzed using the Conserved Domain Database (http://www.ncbi.nlm.nih.gov/Structure/cdd/wrpsb.cgi). The secondary structure of the protein was predicted using Discovery Studio software (Accelrys Software Inc., San Diego, CA). Alignments of multiple DNA and amino acid sequences were generated using ClustalW2 (http://www.ebi.ac.uk/Tools/clustalw2/index.html).

### Heterologous expression of laccase

To construct the expression vectors, YlLac cDNA was amplified using Taq polymerase (Promega), the primers YlLac-f1 and YlLac-r1 (Table 1), and the cDNA with sequence for the native signal peptide sequence as the template. The amplified DNA fragments were digested with *Xho*I and *Xba*I and then subcloned into pPICZαA, resulting in the recombinant plasmid pPICZαA-Sp-YlLac. The primers Wsp*-*YlLac-f1 and Wsp-YlLac-r1 (Table 1) were used to clone laccase cDNA without the sequence for the native signal sequence. The PCR products were digested with *Kpn*I and *Xba*I and then subcloned into pPICZαA, resulting in the recombinant plasmid pPICZαA-Wsp-YlLac. The presence of the PCR products was verified by sequencing. The plasmids pPICZαA-Sp-YlLac and pPICZαA-Wsp-YlLac, and all the expression plasmids were linearized with SacI and transformed into *P*. *pastoris* KM71H competent cells by electroporation with a Genepulser II apparatus (Bio-Rad, Hercules, CA). Transformants containing the YlLac cDNA were selected on yeast extract-peptone-dextrose (YPD) agar plates with 1 M sorbitol and increasing concentration of Zeocin 50–200 μg/ml (Invitrogen). Putative transformants were transferred onto minimal methanol plates containing 0.2 mM CuSO_4_, 0.5 mM ABTS, and 200 μg ml^-1^ Zeocin, and laccase-producing clones were identified after 4 days incubation at 30°C. Laccase-producing transformants were identified by the presence of dark green color appearance around the colonies after four days growth. The high expression transformants which showing a deeper color in the plate were selected for liquid fermentance experiment. The transformed yeast cells were grown in 250 ml flasks containing 50 ml BMGY medium at 250 rpm and 30°C. When the turbidity of the culture reached an optical density of ∼4.0 at 600 nm, the cells were harvested by centrifugation at 4,000×g for 10 min and resuspended in BMMY medium containing 200 μM CuSO_4_ in 250 ml Erlenmeyer flasks. The cultures were grown at 30°C with shaking at 200 rpm, with 0.5% (v/v) methanol being added daily. Every 24 h, 0.5 ml of cultures was sampled from the flasks and laccase activity was determined after separating yeast cells by centrifugation.

### Purification and characterization of recombinant laccase

After 7 days, 1 l of culture was harvested by centrifugation at 4,000×*g* for 20 min, filtered through a 0.22-μm membrane (Millipore, Bedford, MA), and then concentrated by ultrafiltration (Viva-flow, Vivascience, Hannover, Germany) using a 30-kDa cut-off membrane. The fluid was then further concentrated to 5 ml by ultrafiltration using a 10-kDa cutoff membrane (Ultra-4, Amicon, Bedford, MA). This was applied to a DEAE Sepharose TM Fast Flow column (1.6 × 10 cm, Amersham Biosciences, Buckinghamshire, UK) equilibrated with 50 mM sodium acetate buffer (pH 4.8). The column was washed with the same buffer, and absorbed proteins were eluted by a linear concentration gradient of NaCl (0–1 M) at a flow rate of 1 ml min^-1^. The fractions containing laccase activity were pooled, dialyzed, and concentrated to *~*2 ml by ultrafiltration using a 30-kDa cutoff membrane. The concentrated enzyme solution was applied to a Hiload 16/60 Superdex 200 pg column equilibrated with 25 mM sodium acetate containing 15 mM NaCl at pH 5.5. The protein was eluted with the same buffer at a flow rate of 0.5 ml min^-1^. Laccase-rich fractions were pooled and stored at 4°C for further use. The amount of protein in the column effluent was monitored by measuring the absorbance at 280 nm. Chromatographic separation was performed using a BioLogic FPLC system (Bio-Rad, Hercules, CA). All procedures were performed at 4°C. Laccase activity and protein concentrations were determined as described previously [15].

### Enzyme characterization

Sodium dodecyl sulfate polyacrylamide gel electrophoresis (SDS-PAGE; 12% w/v polyacrylamide) was performed as described by Laemmli [19]. The native molecular mass was determined by applying samples to a calibrated Sephacryl S-300 HR 16/60 column as recommended by the supplier (GE Healthcare). Purified recombinant YlLac was treated with endoglycosidase H (NEB, Biolabs UK) according to the manufacturer's protocol. YlLac (6 μg) in 1× glycoprotein denaturing buffer was boiled for 10 min. After cooling, 1 μl of endoglycosidase H was added and the sample was incubated at 37°C overnight. The product of endoglycosidase H treatment was analyzed by SDS-PAGE. Protein bands were stained with Coomassie brilliant blue R-250 (Sigma). The zymogram process was carried out using native PAGE, and the enzyme band was visualized by incubating the gel in 50 mM sodium acetate buffer (pH 4.8) containing 0.1 mM ABTS and 2,6-DMP at room temperature. The UV-vis absorption spectrum of purified laccase was recorded at 25°C on a spectrophotometer (Shimadzu, model UV-1800, Tokyo) in 2 cm path length quartz cells. The effects of pH on laccase activity for various substrates were tested at pH 2–6 in 50 mM citrate-phosphate buffer (pH 2–4) or 50 mM sodium-acetate buffer (pH 4–6). The activity of purified laccase towards the ABTS substrate was examined at 20–80°C at the optimal pH value. The rates of substrate oxidation were determined by measuring the absorbance increases at the respective wavelengths, and the molar extinction coefficients (ε) were obtained from the literature [20]. The effects of various inhibitors and metal ions on the activity of purified laccase were determined using assay mixtures (2 ml) containing appropriately diluted enzyme, 50 mM sodium acetate buffer (pH 4.8), and 0.1 mM ABTS with various concentrations of inhibitors or 5 mM metal ions. Laccase activity in the absence of inhibitor or metal ions was defined as 100%. Measurements were carried out in triplicate. *K*_*m*_ and *V*_*max*_ values of purified YlLac were determined by measuring the activity of the enzyme with various concentrations (0.01–0.1 mM) of ABTS and 2,6-DMP substrates at the optimal condition in each case. Kinetic constants were calculated by the Michaelis-Menten method using the GraphPad Prism 5 program.

### Electrochemical experiments

Electrochemical measurements were carried out using a μAutolab potentiostat from EcoChemie (Utrecht, Netherlands). Cyclic voltammetry (CV) was performed with a platinum disc (working electrode), an SCE reference electrode, and a platinum wire counter electrode. Prior to each voltammetric run, surface cleansing of the working electrode was carried out with alumina according to the manufacturer’s instructions. To determine the redox potentials of laccase T1 centers, a protein redox titration method was employed with potassium octacyanomolybdate (IV and V) mediators. Laccase was placed anaerobically into a cell containing a high concentration of K_3_Mo(CN)_8_ in 50 mM sodium acetate buffer, pH 4.5. Redox potentials were registered with platinum electrodes before and after the addition of enzymes. Further titration was performed with the reduced form of the mediator (K_4_Mo(CN)_8_) in 50 mM sodium acetate buffer, pH 4.5. The voltammogram was obtained with several scan rates (5–25 mV s^-1^) between 0 and 1000 mV. The pH-dependent electrochemical experiments were performed following the same protocol as described in [21] using 50 mM citrate-phosphate buffer (pH 2.0–4) and sodium acetate buffer (pH 4–6).

### Homology modeling, validation, and 2,6-DMP docking

The 3D homology model of YlLac was generated using the Build Homology Models (MODELER) module in Discovery Studio 3.0 (DS 3.0; Accelrys Software Inc., San Diego, CA). The crystal structure of *Trametes* sp. AH28–2 (PDB entry 3KW7) was used as a template. Comparative modeling was performed to generate the most probable structure of the query protein by alignment with the template sequences, simultaneously satisfying spatial restraints and local molecular geometry. Sequence identity between the target and the template was found to be 67% according to BLAST parameters. The fit of the sequence in the current 3D environment for each model was evaluated by the Profile-3D Score/Verify Protein tool as implemented in DS 3.0. In order to confirm the consistency of each model for the docking study, model validation was carried out using PROCHECK as described previously [22,23] and the root mean square deviation (RMSD) was 0.5 Å based on C-alpha atoms. 2,6-DMP was docked into the active site pocket of the YlLac model and *Trametes* sp. AH2B (3KW7) using C-DOCKER, a molecular dynamics (MD) simulated-annealing-based algorithm module from DS 3.0. The protein structure was subjected to energy minimization using the CHARMM forcefield as implemented in DS 3.0. A full potential final minimization was then used to refine the substrate positions.

### Nucleotide sequence accession number

The nucleic acid sequence data reported in this study have been deposited in the GenBank database under accession number JF719545.

### Pretreatment of woody biomass

Woody biomass (*P*. *balsamifera*) was procured from Phygen Co. Ltd. (Daejeon, Korea). The wood chips were milled using a laboratory hammer mill. Milled material was further separated (size reduced to about 2–50 mm) using a portable sieve shaker. The wood chips were screened to remove all particles greater than 35 mm and less than 6 mm in length to ensure smooth operation in disk-milling. The thickness of the accepted chips ranged from 2 to 6 mm. The composition of the *P*. *balsamifera* biomass was 31.4% cellulose, 24.1% hemicellulose, 21% lignin, and 11% ash content on a dry weight basis. 20% (dry weight) woody biomass was suspended in 30 ml 0.5% sulfuric acid (w/w) and held at 121°C for 30 min. The pretreated *P*. *balsamifera biomass* was dried at 100°C until it reached a constant weight before further use [15].

### Phenol removal from pretreated biomass using YlLac

YlLac and a commercial laccase from *Trametes versicolor* (Sigma Aldrich) were used to treat the woody biomass derived from *P*. *balsamifera*. Preliminary assays were performed to optimize the pH, incubation time, and laccase dosage of the phenol removal treatment. Assays were performed in 100-ml Erlenmeyer flasks containing 2 g (dry weight) of pretreated *P*. *balsamifera* biomass in 25 ml of 50 mM buffer (pH 3–6) at 40°C in a rotary shaker (150 rpm) using laccase 5U/ml. Supernatants were periodically analyzed for total phenols by the Folin-Ciocalteau method [24], and the results were expressed as grams of catechol equivalents (CE) per liter of liquid phase.

### Saccharification experiments

A filter paper assay was used to estimate total cellulase activity in the crude enzyme (Celluclast 1.5L, Novozyme, Denmark) and expressed as filter paper units (FPU). The reaction mixtures, which consisted of 0.2 g substrate, and an enzyme loading of 25 FPU, were supplemented with the antibiotics tetracycline (40 μg/mL) and cycloheximide (30 μg/mL) to prevent microbial contamination. The mixture was then incubated at 37°C on a rotary shaker at 150 rpm. Samples were taken from the reaction mixture at different time intervals and immediately heated to 100°C to denature the enzymes. They were then cooled and centrifuged for 10 min at 8000 rpm. Estimation of the total reducing sugar content in the enzymatic hydrolyzate of biomass was done as described previously [15]. The saccharification yield was calculated using the following equation: % saccharification = released reducing sugar (g) × 0.9 × 100/grams of cellulose in substrate, where 0.9 is the mass ratio of anhydroglucose to free glucose [25].

## Results and Discussion

### Cloning of full-length cDNA of the laccase gene

Degenerate primers for conserved CuI (HWHGFFQ) and CuIV (HCHIDFH) regions were designed to amplify the laccase encoding DNA, and a 1523-bp DNA fragment was obtained. When compared with other laccase genes available in the GenBank database, this DNA fragment showed 62% sequence similarity to the laccase gene from *Polyporus ciliatus* (AF176230). Based on this partial sequence, TAIL-PCR was used to obtain the 5′ and 3′ flanking sequences of the laccase fragment. Finally, PCR with specific primers as described in the Materials and Methods section yielded a 1,859-bp complete structural gene encoding the laccase. Sequence analysis revealed that 6 typical fungal introns, with sizes ranging from 48 to 50 bp, interrupted the coding region. All the splicing sites of these introns were in accordance with the GT-AG rule. The 1557-bp full-length cDNA of the laccase gene containing the intact ORF was cloned from *Y*. *lipolytica* SKU507 and designated as YlLac (GenBank accession no. JF719545). The ORF of YlLac encoded a polypeptide with 519 amino acids, including a 19-residue secretion signal peptide (S1 Fig.). The calculated molecular mass of the deduced protein was 52.4 kDa, and the calculated isoelectric point (pI) was 4.86. The YlLac protein contained seven potential N-glycosylation sites (Asn-X-Ser/Thr). The conserved domains of YlLac were predicted, and they were analyzed using the Conserved Domain Database. Multiple alignment of amino acid sequences of YlLac and other laccase proteins were generated with ClustalW2 and it shows that all proteins contain the strictly conserved copper ligand motifs (S1 Fig.). The amino acid residues that act as Cu^2+^ ligands are highly conserved in all blue copper oxidases, including laccases. All of the expected Cu^2+^ ligands (10 His residues and one Cys residue) were present in the YlLac coding sequence and are numbered in S1 Fig., on the basis of whether they coordinate with the type 1, type 2, or type 3 Cu^2+^ centers. The amino acid sequence of YlLac was compared with those of other laccases available in the GenBank database (S1 Fig.). Surprisingly, YlLac was closest to *P*. *ciliatus* (AAG09231) and *T*. *versicolor* (Q12719) laccases with 74% and 71% sequence identity, respectively. A number of phenol-oxidizing enzymes have been cloned and expressed from basidiomycetes fungi [26], bacteria [27], and some actinomyces [28]. However, to our knowledge, this is the first detailed molecular study of such an enzyme characterized from isolated yeast.

### Expression of YlLac in *P*. *pastoris* under different signal peptide

The expression vectors pPICZαA-Sp-YlLac and pPICZαA-Wsp-YlLac with the laccase gene contained the native signal peptide and the α-factor secretion signal peptide, respectively. All clones were under control of the *AOX I* promoter. pPICZαA-Sp-YlLac and pPICZαA-Wsp-YlLac were transformed into *P*. *pastoris* KM71H by electroporation. The positive transformants containing pPICZαA-Wsp-YlLac produced green-halos after 4 days, whereas those containing pPICZαA-Sp-YlLac did not produce green halos after 10 days. No laccase-positive clones were observed from vector control-transformed *P*. *pastoris*. Further studies were carried out using pPICZαA-Wsp-YlLac. The laccase-positive transformants as well as the negative control-pPICZαA were then grown in BMM liquid medium at 30°C and laccase expression was induced by adding 0.5% (v/v) methanol daily. The highest laccase yield from pPICZαA-Wsp-YlLac*-*transformed cells was reached following a 7-days growth at 30°C with maximum laccase activity (1.25U/mL). However, no extracellular laccase activity was detected in culture supernatants of the negative control pPICZαA. The signal peptide is considered an important factor influencing the activity of heterologously expressed laccase [29]. We cloned the native signal peptide of YlLac and the α-factor signal peptide from *P*. *pastoris* into individual yeast expression vectors. The native signal peptide could not direct the active expression of YlLac and α-factor secretion signal peptide efficiently expressed the laccase gene.

### Purification of recombinant YlLac

The production profile of recombinant laccase showed a peak in laccase activity on the 7^th^ day (1290 U/L). Based on this profile, liquid cultures were harvested after 7 days for laccase purification, which consisted of ultrafiltration, DEAE Sepharose TM Fast Flow column purification, and Hiload 16/60 Superdex 200 pg chromatography. Table 2 lists the results after the different steps of laccase purification. Overall, YlLac was purified 94×. The specific activity of the purified enzyme was 263 U mg protein^-1^ (Table 2).

**Table 2:** Purification of YlLac from the culture broth of *P*. *pastoris* KM71H cells.

Purification step	Total protein (mg)	Total Activity (U)	Specific activity (U/mg)	Purification fold	Yield (%)
Crude culture filtrate	456	1290	2.8	1.0	100
Ultrafiltration	305	990	3.2	1.1	76.5
DEAE-cellulose	8.2	582	70.9	25.3	44.9
Gel filtration	1.2	316	263	94.1	11.0

*P*. *pastoris* KM71H was grown for 7 days in BMM medium containing 0.2 mM CuSO_4_ (30°C; 200 rpm). YlLac protein was purified from the culture broth as indicated. The activity was monitored by oxidation of ABTS under standard assay conditions (see materials method). The expression level of YlLac in *P*. *pastoris* was 4.9 mg/L.

**Fig 1:**
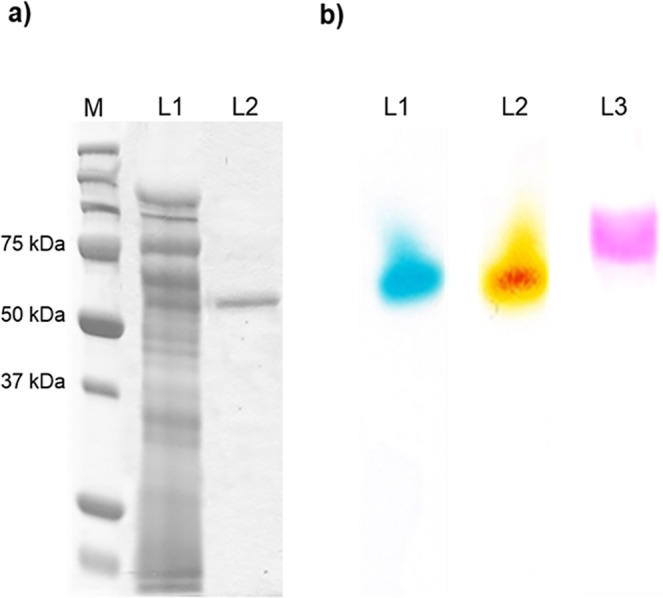
Determination of the molecular mass of purified YlLac by (a) SDS-PAGE of laccase (M, Marker; L1, Crude protein; L2, endoglycosidase-H-treated YlLac); and (b) zymogram activity and glycoprotein staining of the purified laccase enzyme with native PAGE (L1, ABTS; L2, 2,6-DMP; L3, glycoprotein staining). The zymogram process was carried out using native PAGE, and the enzyme band was visualized by incubating the gel in 50 mM sodium acetate buffer (pH 4.8) containing 0.1 mM ABTS and 2,6-DMP at room temperature.

The native molecular mass determined both by gel filtration chromatography on a Sephacryl S-300 HR column and SDS-PAGE revealed that the molecular mass of purified recombinant YlLac was about ~67–68 kDa (data not shown), which was higher than the predicted masses of 54 kDa, indicating the recombinant laccase was glycosylated, similar as other fungal laccases expressed in *P*. *pastoris* [30,31]. After treatment with endoglycosidase H (endoH), the molecular mass of YlLac was reduced to ~55 kDa (Fig. 1a), indicating the recombinant laccase was glycosylated. Glycoprotein and activity staining of the laccase, using ABTS and 2,6-DMP substrates, revealed a single protein band corresponded with activity of the laccase (Fig. 1b).

### Substrate specificity and kinetic property of recombinant YlLac

**Fig 2:**
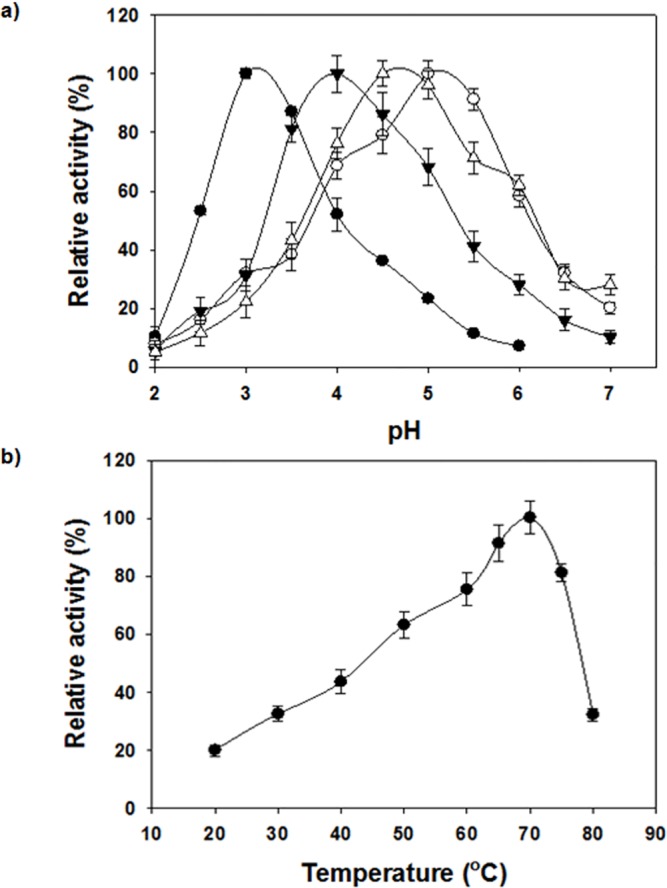
Effect of pH and temperature on YlLac activity. (a) Effect of pH on the activity of purified YlLac towards ABTS (●), toluidine (▼), guaiacol (Δ), and 2,6-DMP (○). Reactions were at room temperature for 3 min in citrate/acetate/phosphate buffer. (b) Effect of temperature on the activity of purified YlLac assessed by the standard assay method. Activities are expressed as percentages of maximum activity; the error bars do not exceed the dimensions of the symbols.

Like other laccases, YlLac oxidized a range of substrates including phenolic and aromatic amine substrates. The purified YlLac oxidized ABTS maximally among the different substrates tested. Substrate oxidization of YlLac is in the following order ABTS > 2,6-DMP > toluidine > l-DOPA > guaiacol > phenyldiamine > 3-aminobenzoic acid (S2 Fig.). No activity was detected with veratryl alcohol. Four substrates were used to determine the effect of pH on YlLac activity. The pH optima obtained for YlLac were in the acidic region (ABTS, pH 3.0; 2,6-DMP, pH 5.0; toluidine, pH 4; guaiacol, pH 4.5), with a sharp decline in enzyme activity as the pH value moved towards the neutral range, reaching an almost undetectable level (Fig. 2a). The optimum temperature for YlLac activity was determined by using ABTS as a substrate, and the maximum activity was observed at 70°C (Fig. 2b).

The stability of purified YlLac was assessed at 60, 65, and 70°C; the enzyme retained at least 50% of its initial activity up to 480 min, 160 min, and 42 min, respectively (S3 Fig.). The kinetic constants of YlLac were determined under optimized conditions using ABTS and 2,6-DMP (S1 Table). The *K*_*m*_ and *V*_*max*_ values of YlLac were found to be 114 μM and 1990 μmol min^-1^ mg^-1^ protein for ABTS, and 75 μM and 1210 μmol min^-1^ mg^-1^ protein for 2,6-DMP, respectively. Catalytic efficiencies (*k*_*cat*_ */K*_*m*_) for ABTS and 2,6-DMP were determined to be 17.5 s^-1^ μM^-1^ and 16.1 s^-1^ μM^-1^, respectively. YlLac exhibits the highest catalytic efficiency (*k*_cat_ */K*_m_) towards ABTS and 2,6-DMP, compared to reported wild-type laccases from various sources (S1 Table). The evolved OB1-laccase mutant showed higher *k*_cat_ */K*_m_ for ABTS than YlLac [2].

### Electrochemical and spectral characteristics of YlLac

**Fig 3:**
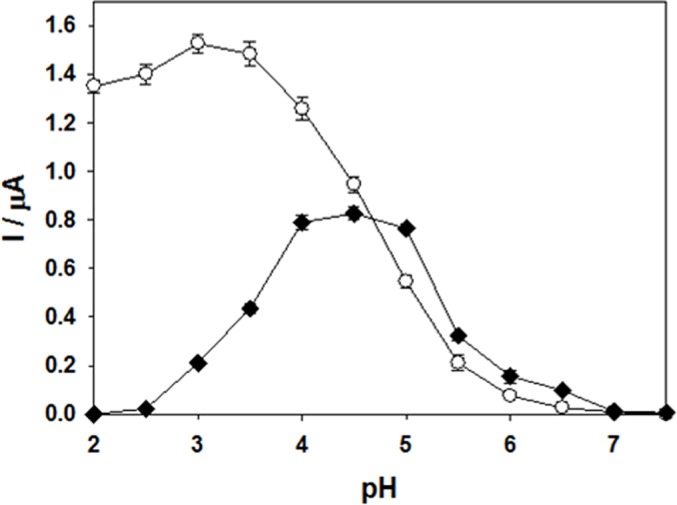
Dependence of the catalytic current on pH for YlLac-coated glassy carbon electrode with ABTS (○) and 2,6-DMP (■), as substrates.

In order to investigate optimal conditions for laccase-mediated electron transfer, we evaluated the effect of pH on the catalytic current. Slow-scan voltammograms (Fig. 3) illustrate the importance of the solution pH on the catalytic current for one non-phenolic mediator (ABTS) and one phenolic mediator (2,6-DMP). Two different trends are clearly observed. For ABTS, the current decreased as the pH of the solution increased, i.e. exhibited a negative slope as pH increased. The optimum pH was around 3. On the other hand, currents recorded with 2,6-DMP exhibited a maximum value in the pH range of 3.5–5 due to proton exchange [32,33].

The purified enzyme has the typical color of blue copper oxidases and contains 4.0 atoms of copper per molecule. The presence of a type 1 copper atom was deduced from the UV/visible spectrum of the purified enzyme (S4 Fig.), which shows a broad peak at 611 nm. In this spectrum, the presence of a shoulder at 333 nm could indicate a binuclear type 2 copper [Cu (II)] complex [34]. The electrochemical analysis for YlLac-1 showed that it has a high redox potential (*E* ^*o*^) of 770 mV for the type 1 Cu (T1) site.

### Effects of inhibitors and metal ions on the activity of recombinant YlLac

A number of compounds were examined for their effects on YlLac activity, including small ions (sodium azide), sulfhydryl group-containing redox reagents (l-cysteine and dithiothreitol, SDS), denaturants (thiourea), and a chelator (EDTA) (S2 Table). Reducing agents such as l-cysteine and dithiothreitol strongly inhibited laccase activity. Enzyme activity was not observed in the presence of 0.1 mM NaN_3_. SDS caused partial inhibition and the metal chelating agent EDTA caused no inhibition. Metal ions such as Cr^3+^, Mg^2+^, and Mn^2+^ inhibited 25%, 24%, and 24% of YlLac activity, respectively. Other metal ions did not show significant inhibition.

### Secondary structure and homology modeling of YlLac

**Fig 4:**
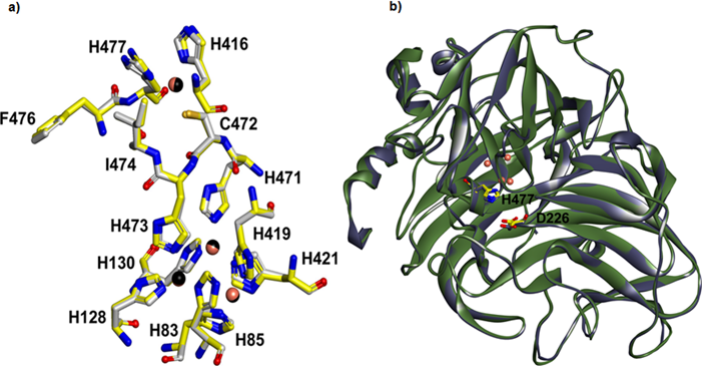
Overall structure of YlLac with its template. (a) Putative catalytic and copper binding domains of YlLac (yellow color carbon) superimposed on *Trametes* sp. AH28–2(PDB entry 3KW7, gray color carbon). The residues are shown in the stick model and are labeled with YlLac amino acid residue numbers. Copper ions for YlLac and *Trametes* sp. AH28–2 are shown in black and metallic sphere, respectively. (b) Ribbon diagram of the superimposed YlLac (green color) and *Trametes* sp. AH28–2 (grey color) structures with the catalytic residues, represented as a stick model. Amino acid numbers are based on the YlLac sequence. The YlLac catalytic site residues (His 477 and Asp 226) corresponding to the *Trametes* sp. AH28–2 catalytic residues have similar orientation. The figure was generated using DS 3.1

The amino acid sequence of YlLac was aligned with that of *Trametes* sp. AH2B (3KW7) using the Align Multiple Sequences module of DS 3.0, and a homology model of YlLac was generated based on the crystal structure of *Trametes* sp. AH2B (3KW7). Four different typically conserved copper-binding domains were predicted based on previous studies [35]. The secondary structure of YlLac is represented schematically in S5 Fig. The catalytic amino acids were compared and analyzed based on the superimposition of the YlLac model onto the template structure 3KW7 (Fig. 4). Upon superimposition, we observed that YlLac and 3KW7 have similar active site environments. The residues in the T1 copper ion site present a planar triangular coordination with two histidines (His416 and His477) and a cysteine (Cys472) (Fig. 4a). The axial position of T1 copper consists of two non-bonding residues, Ile474 and Phe482. The T2 copper center is co-ordinated to His85 and His419, whereas the T3 copper centers are coordinated to 3 histidine residues each (His130, His421, and His471 for T3 (a); and His85, His128, and His473 for T3 (b)). Upon superimposition, we observed that residues His477 and Asp226 of YlLac were corresponding to the proposed catalytic residues (His461 and Asp206) of *Trametes* sp. AH2B laccase (Fig. 4b).

### Substrate docking analysis

**Fig 5:**
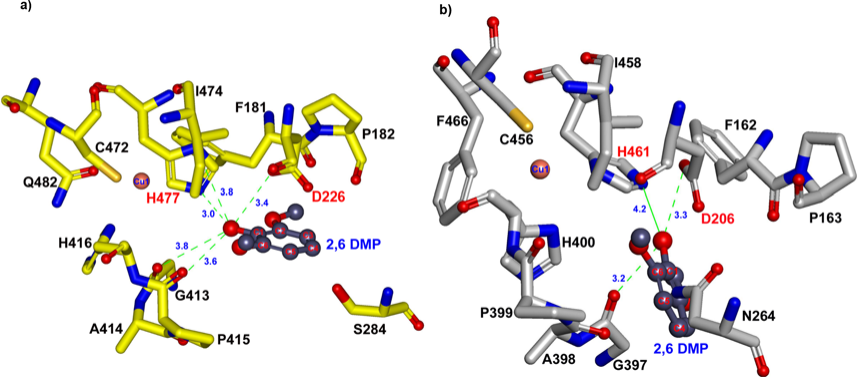
Substrate docking of YlLac with 2,6-DMP in the active site pocket. (a) 2,6-DMP docking into the active site of YlLac. The hydroxyl group of C1 of 2,6-DMP was bound in the active site through H-bonds (green dotted lines) with the oxygen in the carboxyl groups of D226 (3.4 Å) and H477 (3.0 and 3.8 Å). (b) Docking of 2,6-DMP into the active site of 3KW7 (crystal structure). Distances of 3.3 and 4.2 Å have been observed between C1 of 2,6-DMP and nitrogen in D206, respectively. The amino acid residues are shown in a stick model. Catalytic residues are labeled in red, and the residue labels refer to those of the laccase active site.

Based on the homology model of YlLac using the crystal structure of *Trametes* sp. AH2B (3KW7) as a template, the overall shape of the substrate binding pocket (SBP) of YlLac was found to be similar to those of the *Trametes* sp. AH2B and *Trametes trogii* laccases [35]. Homology modeling of the three-dimensional structure indicated that the surface-exposed His477 and Asp226 in the catalytic cavity play major roles in the oxidation of substrates (Fig. 5). The substrate 2,6-DMP bound to the His477 exposed on the surface, and not directly to the T1 copper. His477 has been suggested to be a primary electron acceptor, and it is truly positioned optimally to interact with substrate because of its easy access to the molecule's surface [36]. Another interesting residue in YlLac structure is Asp226 (Fig. 5). This hydrophilic residue in the cavity plays a role in substrate oxidation by accepting a proton from the substrate [36].

According to the in silico analysis the active site residues His477 and Asp226 are conserved in the two structures (YlLac and the 3KW7 template), but hydrogen-bonding differs in these two residues (Fig. 5). In the YlLac structure, the hydroxyl group of C1 of 2,6-DMP was bound in the active site through H-bonds with oxygen in the carboxyl groups of Asp226 (3.4 Å) and His477 (NE2–3.0 Å and ND1–3.8 Å). In contrast, there is one hydrogen bond between the hydroxyl group of C1 of 2,6-DMP and the carboxyl groups of Asp206, with a distance of 3.3 Å, and no H-bond between 2,6-DMP and His461 in the 3KW7 structure. The phenolic substrate 2,6-DMP appears to have a tighter contact through its OH group with the corresponding surface-exposed His477 and Asp226, as compared to the His461 and Asp206 of 3KW7. The hydrogen bonds between the substrate and His477 (3.0 Å and 3.8 Å) exist only in YlLac (Fig. 5). Further, two hydrogen bonds (3.6 Å and 3.8 Å) between G413 and O1 of 2,6-DMP may provide more stability in the YlLac structure compared with that of 3KW7 (3.3 Å). Based on the comparison of the YlLac and 3KW7 structures, these differences might explain the high catalytic efficiency of YlLac. We also investigated, using MD simulation, how slight changes in the substrate-binding microenvironment influence the binding affinity of 2,6-DMP. We calculated the binding free energies (ΔG_bind_) of the receptor/ligand complexes [22,23]. Free energy was calculated for each molecular species (complex, receptor, and ligand), and the ligand-binding free energies were obtained. These calculated binding free energies indicate that the binding of 2,6-DMP to YlLac (ΔG_bind_ = -37.8 kcal/mol) is more favorable than its binding to 3KW7 (ΔG_bind_ = -22.9 kcal/mol).

### YlLac treatment to remove phenol from *P*. *balsamifera* biomass

**Fig 6:**
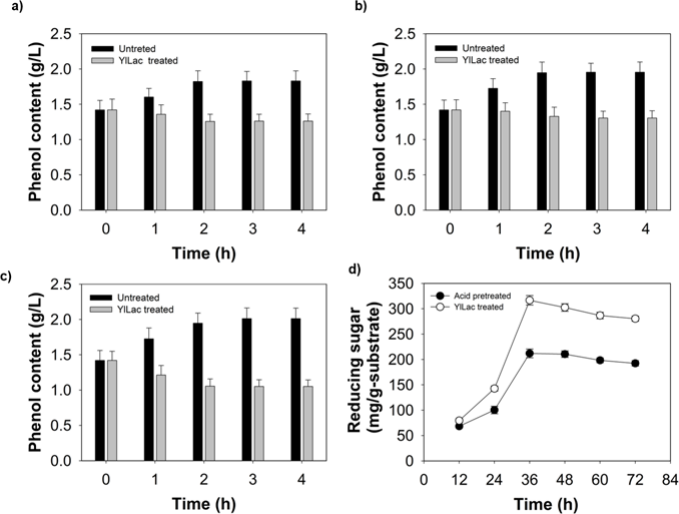
Time course of phenolic content of *P*. *balsamifera* prehydrolysate during pretreatment with YlLac at different pHs, (a) 3, (b) 4, (c) 5. Untreated control (without laccase) is also shown, (d) Reducing sugar production from acid-pretreated *P*. *balsamifera* by Celluclast 1.5L: without laccase pretreatment (black bar), and with YlLac pretreatment (gray bar). Error bars indicate standard deviations from mean values.

Pulverized *P*. *balsamifera* was found to contain 13.8% moisture, 31.4% cellulose, 24.1% hemicellulose, 21.0% lignin, and 10.7% ash. Phenolic compounds in biomass hydrolyzates are known to inhibit saccharifying enzymes and alcohol fermentation [13,14]. In this study, YlLac was used to treat *P*. *balsamifera* biomass to prevent the inhibition of enzymatic cellulolysis by phenolic compounds generated during acid-pretreatment. A spectrophotometric analysis revealed the generation of 2.7 g l^-1^ of total soluble phenolic compounds following acid pretreatment of *P*. *balsamifera* biomass. Treatment with 5 U ml^-l^ of YlLac at pH 3, 4, and 5 removed 40%, 41%, and 62% of phenolic compounds from acid-pretreated biomass, respectively (Fig. 6a, b, c). Subsequently, enzymatic hydrolysis with Celluclast 1.5L was performed at pH 5, the same pH was used for removal of phenol to avoid readjusting the pH condition. Treatment of *P*. *balsamifera* with YlLac before enzymatic hydrolysis yielded 72% saccharification with 316 mg total reducing sugar per gram of substrate. However, untreated (only acid-pretreated) *P*. *balsamifera* yielded 52% saccharification with 212 mg total reducing sugar per gram substrate (Fig. 6d). Treatment of *P*. *balsamifera* with YlLac increased the saccharification yield of *P*. *balsamifera* by 43% (316 mg/g substrate) compared with the untreated sample. The lower saccharification yield from the untreated biomass may have been due to the release of phenolic compounds that can inhibit cell wall-degrading enzymes [13]. Higher saccharification yields after Y1Lac treatment may have been due to a decrease in the unproductive binding of cellulase to lignin after the laccase treatment. YlLac was able to remove phenolic compounds more efficiently than the commercial laccase, leading to a significant increase in the production of reducing sugar from *P*. *balsamifera* by Celluclast 1.5L (Fig. 6d). Further work, such as quantifying and identifying inhibitory phenolic compounds, optimizing the removal of inhibitory compounds, and optimizing hydrolysis conditions, is necessary to explore these findings.

## Conclusions

The YlLac gene encoding the laccase was cloned by TAIL-PCR and successfully overexpressed in *P*. *pastoris*. This is the first report of the cloning and characterization of a laccase gene from yeast. The evidence from enzymology and MD simulation studies strongly suggests that YlLac is a member of the Cu-oxidase superfamily. Compared to other known wild-type laccases, YlLac exhibits the highest catalytic efficiency towards non-phenolic (ABTS) and phenolic (2,6-DMP) substrates, demonstrating its potential for multiple purposes in environmental and industrial applications. Indeed, the removal of free phenolic compounds by YlLac reduced the toxic effects of biomass hydrolyzate and enhanced saccharification yield.

## Supporting Information

S1 Fig Multiple sequence alignment of YlLac with other fungal laccases. The accession numbers are: ABN13592 (*Polyporus brumalis*), ACR50978 (*Coriolopsis gallica*), AAM18408 (*Trametes pubescens*), BAD98307 (*Trametes versicolor*), AAG09231 (*Polyporus ciliatus*). The numbers 1, 2 and 3 corresponds to the co-ordination sites for the types 1, 2 and 3 coppers. The underline indicates the 19-residues for secretion signal peptide. Residue positions identical in all six sequences are indicated with gray color. The CLUSTAL X algorithm was used for alignment. The YlLac catalytic site residues Asp226 (▲) and His477 (Δ) are also indicated.
(TIF)

S2 Fig Substrate oxidizing activity of purified YlLac. The activity was determined relative to ABTS (100%). Assays were carried out in 50 mM sodium acetate buffer (pH 4.8). Absorbance (A) and the molar extinction coefficients (ε_max_) were obtained from the literature [20], ABTS (A_420_, ε_max_ 39000 M^−1^ cm^−1^); 2, 6-DMP (A_470_, ε_max_ 35600 M^−1^ cm^−1^); Toluidine (A_366_, є_max_ 35600 M^−1^ cm^−1^); l-DOPA (A_460_, є_max_ 38000 M^−1^ cm^−1^); Guaiacol (A_436_, ε_max_ 6400 M^−1^ cm^−1^); Phenyldiamine (A_515_, ε_max_ 43100 M^−1^ cm^−1^); 3-Aminobenzoic acid (A_410_, ε_max_ 29000 M^−1^ cm^−1^).
(TIF)

S3 Fig Thermal stability profiles of purified YlLac. Thermal stability profiles of purified YlLac in the presence of 0.1 mM ABTS at 60°C (○), 65°C (▼), and 70°C (Δ). Residual activity was measured under standard conditions.
(TIF)

S4 Fig UV/vis spectrum of YlLac. YlLac shows a laccase-typical absorption spectrum. The maximum at 592 nm corresponds to type I or blue copper and the shoulder around 330 nm is characteristic for type 3 copper centers.
(TIF)

S5 Fig The sequence alignment between YlLac and its template structure *Trametes* sp. AH28–2(PDB entry 3KW7). The sequence identity is 67% and the sequence similarity is 76%. The secondary structure cartoon shown is based on the Kabsch and Sander method. The secondary structure elements of YlLac are color coded, with helices in red, strands in blue.
(TIF)

S1 Table Kinetic parameters of YlLac compared to those of selected laccase enzymes. (DOCX)

S2 Table Effect of typical laccase inhibitors on the YlLac activity. (DOCX)
