# Supplementary figures and images for: A Highly Efficient Recombinant Laccase from the Yeast Yarrowia lipolytica and Its Application in the Hydrolysis of Biomass
Source: PLoS One. 2015 Mar 17;10(3):e0120156. doi: 10.1371/journal.pone.0120156 (PMC4363317; doi:10.1371/journal.pone.0120156)

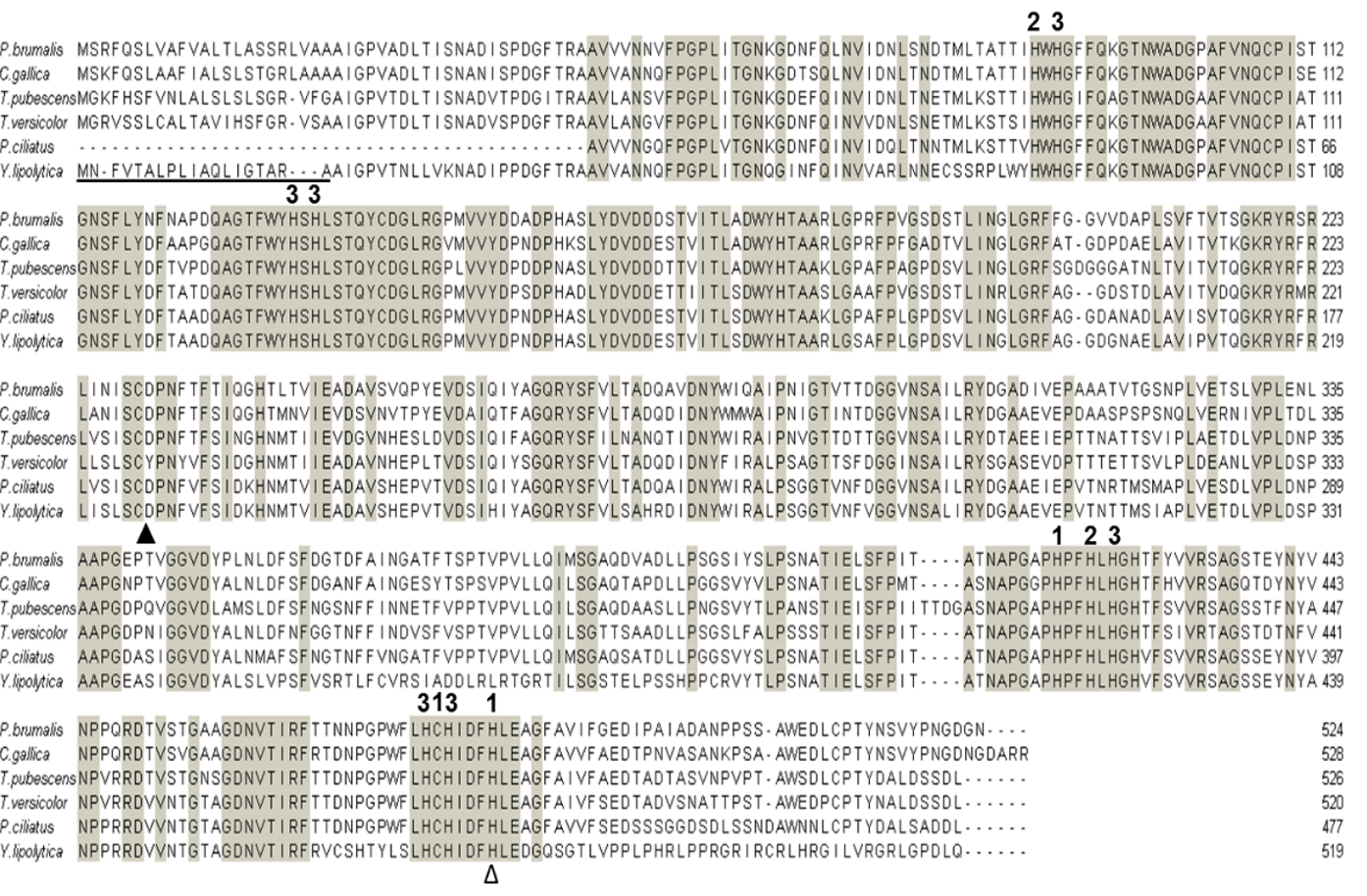

Supplement: S1 Fig — The accession numbers are: ABN13592 (Polyporus brumalis), ACR50978 (Coriolopsis gallica), AAM18408 (Trametes pubescens), BAD98307 (Trametes versicolor), AAG09231 (Polyporus ciliatus). The numbers 1, 2 and 3 corresponds to the co-ordination sites for the types 1, 2 and 3 coppers. The underline indicates the 19-residues for secretion signal peptide. Residue positions identical in all six sequences are indicated with gray color. The CLUSTAL X algorithm was used for alignment. The YlLac catalytic site residues Asp226 (▲) and His477 (Δ) are also indicated. (TIF) [file pone.0120156.s001.tif]

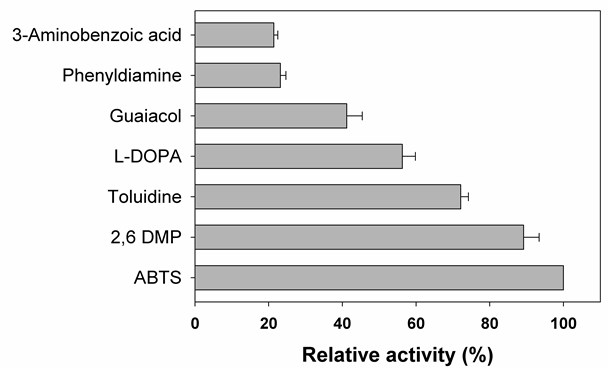

Supplement: S2 Fig — The activity was determined relative to ABTS (100%). Assays were carried out in 50 mM sodium acetate buffer (pH 4.8). Absorbance (A) and the molar extinction coefficients (εmax) were obtained from the literature [20], ABTS (A420, εmax 39000 M−1 cm−1); 2, 6-DMP (A470, εmax 35600 M−1 cm−1); Toluidine (A366, єmax 35600 M−1 cm−1); l-DOPA (A460, єmax 38000 M−1 cm−1); Guaiacol (A436, εmax 6400 M−1 cm−1); Phenyldiamine (A515, εmax 43100 M−1 cm−1); 3-Aminobenzoic acid (A410, εmax 29000 M−1 cm−1). (TIF) [file pone.0120156.s002.tif]

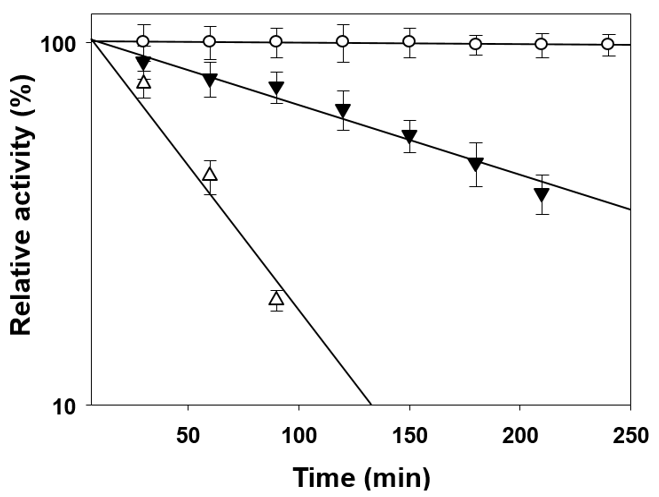

Supplement: S3 Fig — Thermal stability profiles of purified YlLac in the presence of 0.1 mM ABTS at 60°C (○), 65°C (▼), and 70°C (Δ). Residual activity was measured under standard conditions. (TIF) [file pone.0120156.s003.tif]

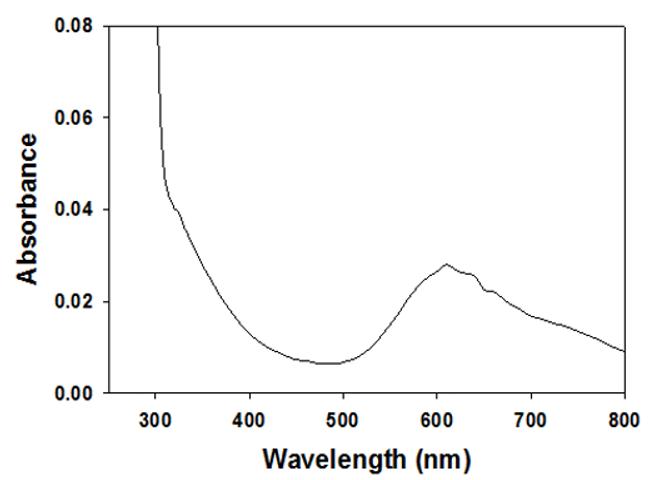

Supplement: S4 Fig — YlLac shows a laccase-typical absorption spectrum. The maximum at 592 nm corresponds to type I or blue copper and the shoulder around 330 nm is characteristic for type 3 copper centers. (TIF) [file pone.0120156.s004.tif]

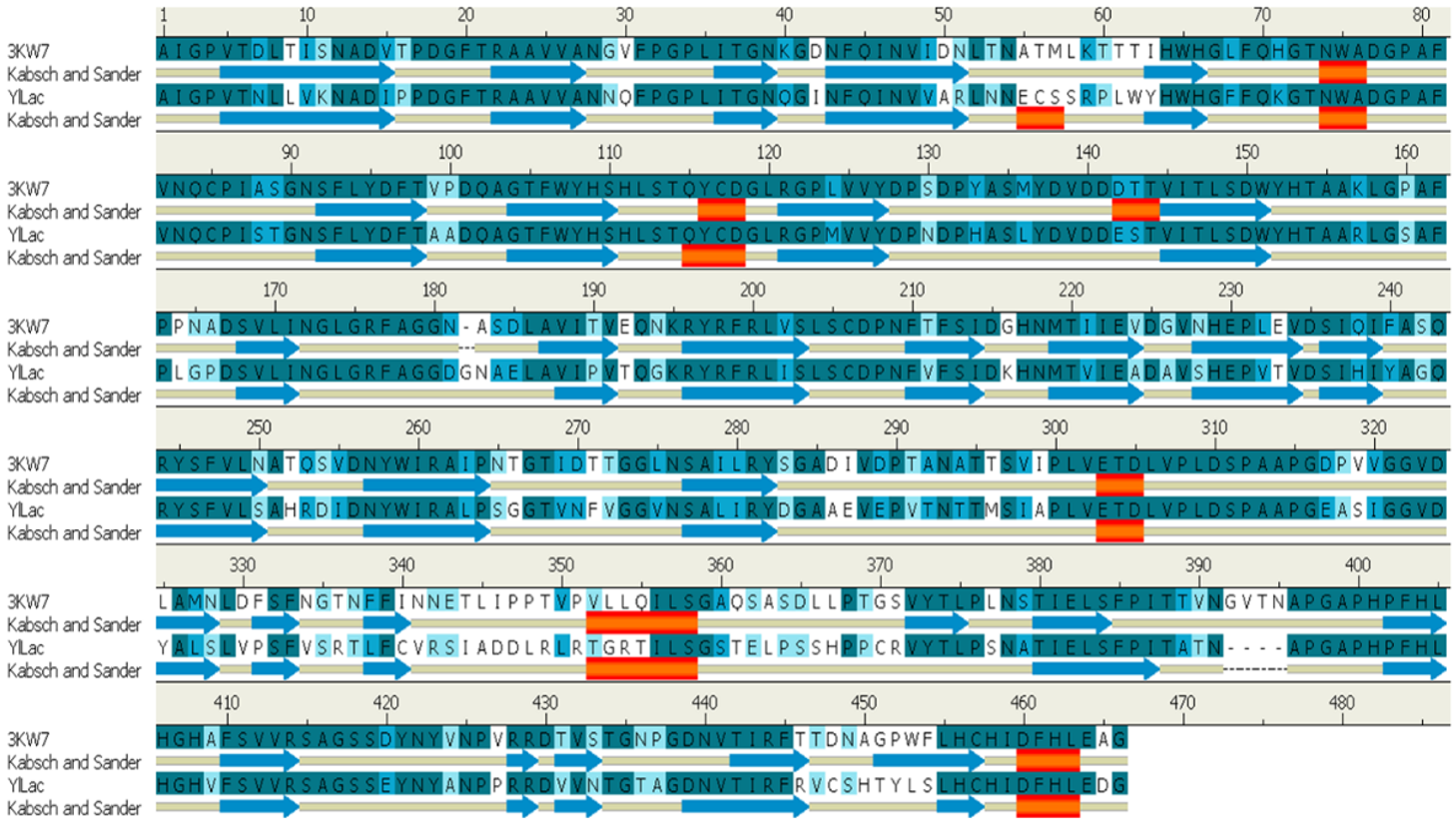

Supplement: S5 Fig — The sequence identity is 67% and the sequence similarity is 76%. The secondary structure cartoon shown is based on the Kabsch and Sander method. The secondary structure elements of YlLac are color coded, with helices in red, strands in blue. (TIF) [file pone.0120156.s005.tif]
