# Supplementary material for: A Highly Efficient Recombinant Laccase from the Yeast Yarrowia lipolytica and Its Application in the Hydrolysis of Biomass
Source: PLoS One. 2015 Mar 17;10(3):e0120156. doi: 10.1371/journal.pone.0120156 (PMC4363317; doi:10.1371/journal.pone.0120156)
Supplement: S1 Table — (DOCX) [file pone.0120156.s006.docx]

**S1Table.** Kinetic parameters of YlLac compared to those of selected laccase enzymes.

| Microbial strain | ABTS | | | 2,6-DMP | | | Reference |
| --- | --- | --- | --- | --- | --- | --- | --- |
|  | *K*_m_  (µM) | *k*_cat_  (s^–1^) | *k*_cat_*/K*_m_  (s^–1^ µM^–1^) | *K*_m_  (µM) | *k*_cat_  (s^–1^) | *k*_cat_*/K*_m_  (s^–1^ µM^–1^) |  |
| *Bacillus subtilis* | 124 | 322 | 2.5 | 216 | 29 | 0.1 | [1] |
| *Trametes* sp. AH28-2 | 177 | 2280 | 12.9 | 109 | 189 | 1.7 | [2] |
| *Pycnoporus sanguineus* | 77.0 | 68.0 | 0.9 | 203 | 6.92 | 0.03 | [3] |
| *Panus tigrinus* CBS 577.79 | 31.0 | 185 | 5.9 | 119 | 366 | 3.0 | [4] |
| *Phoma sp.* UHH 5-1-03 | 8 | 10.6 | 1.3 | 266 | 4.3 | 0.01 | [5] |
| *Melanocarpus albomyces* | 400 | 28.3 | 0.07 | 11 | 10.1 | 0.9 | [6] |
| *Fusarium solani* MAS2 | 79.4 | 107 | 1.3 | 8.6 | 121 | 1.4 | [7] |
| *Scytalidium thermophilum* | 260 | 1431 | 5.5 | 360 | 3495 | 9.7 | [8] |
| *Trametes versicolor* | 169 | 125 | 0.7 | 249 | 49 | 0.1 | [9] |
| *Yarrowia lipolytica* (native) | 115 | 2215 | 19.3 | 76 | 985 | 13.0 | [10] |
| *Yarrowia lipolytica* (recombinant) | 114 | 1990 | 17.5 | 75 | 1210 | 16.1 | This study |

**Reference**

1. Martins LÌgO, Soares CuM, Pereira MM, Teixeira M, Costa T, et al. (2002) Molecular and biochemical characterization of a highly stable bacterial laccase that occurs as a structural component of the *Bacillus subtilis* endospore coat. J Biol Chem 277: 18849-18859.

2. Xiao YZ, Chen Q, Hang J, Shi YY, Wu J, et al. (2004) Selective induction, purification and characterization of a laccase isozyme from the basidiomycete *Trametes* sp. AH28-2. Mycologia 96: 26-35.

3. Lu L, Zhao M, Zhang BB, Yu SY, Bian XJ, et al. (2007) Purification and characterization of laccase from *Pycnoporus sanguineus* and decolorization of an anthraquinone dye by the enzyme. Appl Microbiol Biotechnol 74: 1232-1239.

4. Litthauer D, van Vuuren MJ, van Tonder A, Wolfaardt FW (2007) Purification and kinetics of a thermostable laccase from *Pycnoporus sanguineus* (SCC 108). Enzyme Microb Tech 40: 563-568.

5. Junghanns C, Pecyna MJ, Bohm D, Jehmlich N, Martin C, et al. (2009) Biochemical and molecular genetic characterisation of a novel laccase produced by the aquatic ascomycete Phoma sp. UHH 5-1-03. Appl Microbiol Biotechnol 84: 1095-1105.

6. Kallio JP, Auer S, Jänis J, Andberg M, Kruus K, et al. (2009) Structure-function studies of a *Melanocarpus albomyces* laccase suggest a pathway for oxidation of phenolic compounds. J Mol Biol 392: 895-909.

7. Wu YR, Luo ZH, Kwok-Kei Chow R, Vrijmoed LL (2010) Purification and characterization of an extracellular laccase from the anthracene-degrading fungus *Fusarium solani* MAS2. Bioresour Technol 101: 9772-9777.

8. Younes SB, Sayadi S (2011) Puriﬁcation and characterization of a novel trimeric and thermotolerant laccase produced from the ascomycete *Scytalidium thermophilum* strain. J Mol Catal B: Enzym 73: 35-42.

9. Theerachat M, Emond S, Cambon E, Bordes F, Marty A, et al. (2012) Engineering and production of laccase from *Trametes versicolor* in the yeast *Yarrowia lipolytica*. Bioresour Technol 125: 267-274.

10. Lee KM, Kalyani D, Tiwari M, Kim TS, Dhiman SS, et al. (2012) Enhanced enzymatic hydrolysis of rice straw by removal of phenolic compounds using a novel laccase from yeast *Yarrowia lipolytica*. Bioresour Technol 123: 636-645.
