# Supplementary material for: A Highly Efficient Recombinant Laccase from the Yeast Yarrowia lipolytica and Its Application in the Hydrolysis of Biomass
Source: PLoS One. 2015 Mar 17;10(3):e0120156. doi: 10.1371/journal.pone.0120156 (PMC4363317; doi:10.1371/journal.pone.0120156)
Supplement: S2 Table — (DOCX) [file pone.0120156.s007.docx]

**S2 Table.** Effect of typical laccase inhibitors on the YlLac activity. Relative activities (%) were measured using ABTS as substrate after adding each inhibitor to assay mixture (purified YlLac in 50 mM sodium acetate buffer pH 4.8) to reach the final concentrations of inhibitor.

| Compound | Concentration (mM) | Relative activity (%) |
| --- | --- | --- |
| None | - | 100 |
| Sodium azide | 0.1 | 0 |
| l-Cysteine | 0.1 | 76.6 ± 2.1 |
|  | 0.5 | 11.1 ± 1.5 |
|  | 1 | 0 |
| Dithiothreitol | 0.1 | 61.1 ± 2.4 |
|  | 0.5 | 2.0 ± 0.4 |
|  | 1 | 0 |
| Thiourea | 0.1 | 91.1 ± 2.1 |
|  | 0.5 | 42.2 ± 2.0 |
|  | 1 | 12.3 ± 1.5 |
| SDS | 0.1 | 97.4 ± 2.4 |
|  | 0.5 | 82.5 ± 3.1 |
|  | 1 | 63.3 ± 2.0 |
| EDTA | 0.1 | 100 |
|  | 0.5 | 100 |
|  | 1 | 100 |
